# Iron and Zinc in the Embryo and Endosperm of Rice (*Oryza sativa* L.) Seeds in Contrasting 2′-Deoxymugineic Acid/Nicotianamine Scenarios

**DOI:** 10.3389/fpls.2018.01190

**Published:** 2018-08-21

**Authors:** Pablo Díaz-Benito, Raviraj Banakar, Sara Rodríguez-Menéndez, Teresa Capell, Rosario Pereiro, Paul Christou, Javier Abadía, Beatriz Fernández, Ana Álvarez-Fernández

**Affiliations:** ^1^Department of Plant Nutrition, Estación Experimental de Aula Dei, Consejo Superior de Investigaciones Científicas, Zaragoza, Spain; ^2^Departament de Producció Vegetal i Ciència Forestal, Universitat de Lleida-Agrotecnio Center, Lleida, Spain; ^3^Department of Physical and Analytical Chemistry, Faculty of Chemistry, University of Oviedo, Oviedo, Spain; ^4^ICREA, Catalan Institute for Research and Advanced Studies, Barcelona, Spain

**Keywords:** metals, laser ablation, ligands, mass spectrometry, rice, seeds

## Abstract

Iron and Zn deficiencies are worldwide nutritional disorders that can be alleviated by increasing the metal concentration of rice (*Oryza sativa* L.) grains *via* bio-fortification approaches. The overproduction of the metal chelator nicotianamine (NA) is among the most effective ones, but it is still unclear whether this is due to the enrichment in NA itself and/or the concomitant enrichment in the NA derivative 2′-deoxymugineic acid (DMA). The endosperm is the most commonly consumed portion of the rice grain and mediates the transfer of nutrients from vegetative tissues to the metal rich embryo. The impact of contrasting levels of DMA and NA on the metal distribution in the embryo and endosperm of rice seeds has been assessed using wild-type rice and six different transgenic lines overexpressing nicotianamine synthase (*OsNAS1*) and/or barley nicotianamine amino transferase (*HvNAATb*). These transgenic lines outlined three different DMA/NA scenarios: (i) in a first scenario, an enhanced NA level (*via* overexpression of *OsNAS1*) would not be fully depleted because of a limited capacity to use NA for DMA synthesis (lack of -or low- expression of *HvNAATb*), and results in consistent enrichments in NA, DMA, Fe and Zn in the endosperm and NA, DMA and Fe in the embryo; (ii) in a second scenario, an enhanced NA level (*via* overexpression of *OsNAS1*) would be depleted by an enhanced capacity to use NA for DMA synthesis (*via* expression of *HvNAATb*), and results in enrichments only for DMA and Fe, both in the endosperm and embryo, and (iii) in a third scenario, the lack of sufficient NA replenishment would limit DMA synthesis, in spite of the enhanced capacity to use NA for this purpose (*via* expression of *HvNAATb*), and results in decreases in NA, variable changes in DMA and moderate decreases in Fe in the embryo and endosperm. Also, quantitative LA-ICP-MS metal map images of the embryo structures show that the first and second scenarios altered local distributions of Fe, and to a lesser extent of Zn. The roles of DMA/NA levels in the transport of Fe and Zn within the embryo are thoroughly discussed.

## Introduction

The deficiencies of iron (Fe) and zinc (Zn) are among the most important nutritional disorders in plants and humans. These elements play key roles as cofactors and structural components (e.g., Fe in cytochromes and Zn in Zn-finger proteins, respectively) in many proteins. Near 33% of world human population is affected by *ferropenic anemia*, a low red blood cell count due to Fe deficiency (McLean et al., 2009), whereas Zn deficiency causes about 1.5% of all deaths and about 20% of the perinatal mortality worldwide (Nriagu, 2007). Furthermore, a potential outcome of both metal deficiencies is neuropsychological impairment (Sandstead, 2000). Many of these cases of malnutrition could be solved with a diet enriched in Fe and Zn (De Benoist et al., 2008; White and Broadley, 2009). Foods rich in micronutrients such as meat and vegetables, unlike staple foods, are expensive and cannot be stored for long periods. Since rice is a staple food in large areas of the world, particularly in underdeveloped regions, biofortification of rice grains with Fe and Zn is a realistic target to alleviate these nutritional disorders. In most parts of the world, rice is traditionally cooked after milling and polishing, reducing the nutritional value because of the removal of the metal-rich bran and embryo, with only the endosperm remaining. Also, rice can be treated hydrothermally (parboiling) prior to milling to reduce breakage, increasing the nutritional value because of micronutrient transport from bran to endosperm, although changes of color, odor and texture, as well as mycotoxin risks, may arise (Rohman et al., 2014). Conventional, agronomic and transgenic approaches have been used for rice biofortification (reviewed by Garg et al., 2018), with approaches based on molecular genetics having the advantage that any gene with a demonstrated utility may be further used for targeting the biofortification of other crops. Furthermore, whereas diet diversification might be an option in principle, in practical terms poor people in developing counties cannot afford a diverse diet. Therefore, a major challenge for biofortification strategies in rice is to increase the concentrations of Fe and Zn in the endosperm.

Rice plants take up Fe from the soil using mechanisms classically ascribed to Strategies I and II (Ishimaru et al., 2006). Strategy I, used by non-graminaceous species, involves the uptake of Fe(II) *via* a Fe-Regulated Transporter (IRT) (Vert et al., 2002). Rice roots do express *OsIRT1* and this transporter is strongly up-regulated upon Fe deficiency (Ishimaru et al., 2006). In Strategy II, used by Gramineae, Fe acquisition is mediated by the synthesis and secretion of phytosiderophores (PSs) of the mugineic acid family (MAs) (Kobayashi et al., 2006). The synthesis of MAs starts from the condensation of three *S*-adenosyl methionine molecules to produce nicotianamine (NA) *via* nicotianamine synthase (NAS). Then, 2′-deoxymugineic acid (DMA) is synthesized from NA *via* nicotianamine aminotransferase (NAAT) and DMA synthase. In response to Fe deficiency, rice roots synthesize DMA (Takagi, 1993), which is secreted to the rhizosphere *via* TOM1 (Transporter Of Mugineic acid 1) (Nozoye et al., 2011). The secreted DMA is able to solubilize sparingly soluble Fe(III) by forming Fe(III)-DMA complexes, which are then taken up by root cells *via* transporters of the YSL (Yellow Stripe 1-Like) family (Curie et al., 2009). Zinc is usually taken up by plants as the free Zn(II) ion by root epidermal cells (Ishimaru et al., 2011; Sinclair and Krämer, 2012). Also, PSs can form Zn complexes that are as stable as Fe(III)-PS (Murakami et al., 1989), and both the secretion of PS and uptake of Zn-PS *via* YSL transporters have been observed in grasses (von Wirén et al., 1996; Suzuki et al., 2006; Widodo et al., 2010).

The short- and long-distance transport of Fe and Zn in grasses occurs both as free ions and metal complexes. Different PS-metal complexes have been found in plant fluids, including Fe(III)-DMA and Zn(II)-DMA in the xylem sap of wheat (Xuan et al., 2006), and Fe(III)-DMA and Zn(II)-NA in the phloem sap of rice (Nishiyama et al., 2012). Since both plant fluids transport nutrients from maternal to filial tissues at the reproductive stage (Waters and Grusak, 2007), Fe and Zn in grains can originate either from a direct root-to-seed route *via* xylem or from the remobilization of Fe from old and senescing leaves *via* phloem (Grillet et al., 2014; Yoneyama et al., 2015). An internal transport of these metals also occurs once they are in the grain, since the developing embryo is a sink for nutrients and the endosperm constitutes a nutrient reservoir. A complex network of transporters belonging to different families mediates the movement of both metals within cells and the whole plant; some proteins (e.g., IRT, P1B-type heavy metal ATPases) are capable of transporting Zn and Fe divalent ions (Takahashi et al., 2012; Kolaj-Robin et al., 2015), whereas others are capable of transporting Zn and Fe complexes (e.g., YSL family transport metal-NA or metal-PSs complexes; Curie et al., 2009).

The fact that mutations in the genes involved in NA and DMA synthesis and those of YSL transporters do not cause substantial decreases in the seed Fe concentrations (e.g., *osnas3*, Lee et al., 2009b) supports that metal transport systems in plants are redundant. On the other hand, the fact that the overexpression of genes involved in DMA/NA synthesis and those of YSL transporters only lead to limited increases in seed Fe concentrations (Banakar et al., 2017a,b) support the existence of regulatory feedback loops. Once in the rice grain, Fe may be stored in ferritins to avoid toxicity (Briat et al., 2010), sequestered in vacuoles (Kim et al., 2006) or bound to phytate, a P-rich molecule preferentially located in the aleurone layer (Persson et al., 2009). Phytate is also considered to control Zn levels in seeds (Raboy, 2003), and has a low bioavailability during human digestion (Guttieri et al., 2004; Gupta et al., 2015).

A range of transgenic approaches have been used to increase micronutrient concentrations in rice grains (Bashir et al., 2013; Nozoye, 2018), including: (i) increases in the expression of NA, DMA, YSL and ferritin synthesis genes; (ii) increases in absorption promoters, and/or (iii) decreases in inhibitors of absorption in human gut. The highest increase achieved so far in the concentrations of Fe and Zn in polished seeds has been achieved with an *OsNAS2-SoyferH-1* construct, leading to 6- and 4-fold increases, respectively, over the WT values (Trijatmiko et al., 2016). Several studies have found a positive effect of increasing NA synthesis in achieving rice biofortification with Fe and Zn (Nozoye, 2018), but it is still unclear whether this is due to the enrichment in NA itself and/or the concomitant enrichment in the NA derivative DMA. Moreover, most previous studies have focused on the grain endosperm, with the embryo, a part of the seed of outmost importance for seed formation, germination and viability, being studied in less detail.

In this work, the impact of contrasting levels of NA and DMA on the distribution of metals in the embryo and endosperm of rice seeds has been studied, using wild-type (WT) rice and six transgenic lines overexpressing *OsNAS1* and/or expressing barley *NAAT* (*HvNAATb*). Increasing only DMA led to Fe enrichments in the embryo and endosperm, whereas increasing DMA in combination with NA produced Fe and Zn enrichments in both tissues. Laser ablation inductively coupled plasma mass spectrometry (LA-ICP-MS) was also used to determine the spatial localization and concentrations of Fe, Zn and other elements within the embryo structures, providing the first quantitative set of data for these metals in the embryo tissues of biofortified rice seeds. We discuss the changes induced in the elemental distribution in contrasting DMA/NA scenarios, providing new insights into the Fe and Zn transport mechanisms within the embryo.

## Materials and Methods

### Plant Material

Rice plants (*Oryza sativa* L. cv EYI 105) were transformed to obtain genotypes overexpressing *OsNAS1* and/or *HvNAATb* genes and therefore with high levels of NA and/or DMA. The details of the cloning, expression and transformation were described in detail in Banakar et al. (2017b). The six lines used are two overexpressing *OsNAS1* alone (N1 and N2), two expressing *HvNAATb* alone (D1 and D2) and two expressing *OsNAS1* and *HvNAATb* together (ND1 and ND2). Lines ND1 and ND2 were also used, although in different growth conditions, in Banakar et al. (2017b).

#### Gene Cloning

The cDNAs of *OsNAS1* (GenBank ID: AB021746.2, 999 bp) and/or *HvNAATb* (GenBank ID: AB005788.1, 1,656 bp) were cloned from roots of rice (*O. sativa* cv EYI 105) and barley (*Hordeum vulgare* L. cv Ordalie) grown *in vitro* on MS medium without Fe (Murashige and Skoog, 1962) for 2 weeks. Total RNA was extracted with RNeasy Plant Mini kit (Qiagen, Hilden, Germany) and 1 μg of RNA was used for reverse transcription using Omniscript RT Kit (Qiagen) by RT-PCR. The full-size cDNAs for *OsNAS1* (999 bp) and *HvNAATb* (1,656 bp) were amplified by PCR using the primer combinations *OsNAS1*-BamHI-FOR (5′-AGG ATC CAT GGA GGC TCA GAA CCA AGA GGT CG-3′) and *OsNAS1*-HindIII-REV (5′-AAA GCT TCA TAA TAT AGT GCG CCT GAT CGT CCG GCT GT-3′), and *HvNAATb*-BamHI-FOR (5′-AGG ATC CAT GGC CAC CGT ACG GCC AGA GAG CGA CG-3′) and *HvNAATb*-HindIII-REV (5′-AAA GCT TCT AGC AAT CAT CGC TCG CTC GAA TTT CTC-3′), respectively. The products were transferred to the pGEM-T Easy vector (Promega, Madison, Wisconsin, USA) for sequencing and verification. The *OsNAS1* and *HvNAATb* cDNAs were inserted at the BamHI and HindIII sites of expression vector pAL76 (Christensen and Quail, 1996), which contains the maize UBI1 promoter and first intron, and an *Agrobacterium tumefaciens nos* transcriptional terminator. A separate vector harboring the constitutive cauliflower mosaic virus 35S promoter (CaMV35S) was used to provide the hygromycin phosphotransferase (*hpt*) selectable marker (Christou and Ford, 1995).

#### Rice Transformation and Growth Conditions

Rice embryos were isolated from *Oryza sativa* L. (cv EYI 105) mature seeds and grown on MS medium containing 2.5 mg L^-1^ 2,4-dichlorophenoxyacetic acid (2,4-D) as in Sudhakar et al. (1998). After 7 days, embryos were incubated on high-osmoticum MS medium (0.2 M mannitol, 2.5 mg L^-1^ 2,4-D) for 4 h (Sudhakar et al., 1998; Valdez et al., 1998), and then bombarded with gold particles carrying the transgenes and the *hpt* selectable marker (Christou et al., 1991). Bombarded embryos were selected on MS medium supplemented with 2.5 mg L^-1^ 2,4-D and 30 mg L^-1^ hygromycin, and callus pieces were transferred sequentially to shooting and rooting medium containing hygromycin (Sudhakar et al., 1998; Valdez et al., 1998).

Regenerated plantlets were transferred to pots filled with substrate (Traysubstract, Klasmann-Deilmann GmbH, Geeste, Germany) and grown flooded in large trays in growth chambers at 26°C, 900 μmol m^-2^ s^-1^ PPFD PAR with a 12/12 h light/dark regime and 80% relative humidity. Plants were watered with 100 μM Fe(III)-EDDHA (Sequestrene 138 Fe G-100; Syngenta Agro SA, Madrid, Spain) until flowering, and then self-pollinated. The Fe(III)-EDDHA solution in the trays was replaced every week. T_0_ plants were grown to maturity, T_1_ seeds were harvested and the resulting plants were crossed over two generations to generate a homozygous T_3_ population. T_3_ seeds from the transgenic lines were germinated on ½ MS medium containing 30 mg L^-1^ hygromycin, whereas WT seeds were germinated on ½ MS medium without hygromycin. Five-day-old seedlings from wild type and transgenic lines were transferred to pots filled with substrate as described above, and maintained until the T_4_ seeds had matured. Sampling of the T_3_ flag leaf was performed to confirm the expression of the genes of interest and T_4_ seeds were harvested to study the metal quantitative distribution in different seed tissues.

The anatomical denominations in this study comply with the monograph by Hoshikawa (1989). Grains from the same panicle were harvested, and some of them were stored at 4°C until metal localization analyses and others de-husked by hand, to avoid metal contamination from the de-husking machine. Then, brown seeds were separated into embryo and endosperm, in both cases maintaining the corresponding aleurone layer, using new stainless steel razor blades and binocular magnifying glasses. For each genotype, embryo and endosperm samples were obtained pooling materials from 50–100 and 10 seeds, respectively, and 3–4 replications were used. Samples were ground in liquid N_2_ with ceramic mortar and pestle until a fine powder was obtained, and aliquots were stored at -80°C until analysis.

### RNA Blot Analysis

Total RNA was isolated from the flag leaf (T_3_ generation) using the RNeasy Plant Mini Kit (Qiagen), and 20-μg aliquots were fractionated on a denaturing 1.2% agarose gel containing formaldehyde before blotting. The membranes were probed with digoxigenin-labeled partial *OsNAS1* or *HvNAATb* cDNAs at 50°C overnight, using DIG Easy Hyb (Roche Diagnostics, Mannheim, Germany). After washing and immunological detection with anti-DIG-AP (Roche Diagnostics) according to the manufacturer’s instructions, CSPD chemiluminescence (Roche Diagnostics) was detected on Kodak BioMax light film (Sigma–Aldrich, St Louis, MO, United States).

### Analysis of Metals, Nicotianamine and 2′-Deoxymugineic Acid in the Embryo and Endosperm

Fifty mg of ground embryo or endosperm tissue from grains of the same panicle were dried at 60°C and digested with ultrapure 21% HNO_3_ (Trace Select Ultra, Fluka) and 6% H_2_O_2_ (Suprapur, Merck) for 55 min at 190°C in an Ethos 1 microwave oven (Milestone Srl., Sorisole, Italy). Digests were analyzed (3 independent replicates) for Fe, Mn, Cu, and Zn by inductively coupled plasma mass spectrometry (ICP-MS), using an Agilent 7500ce (Agilent, Santa Clara, CA, United States) and monoelemental standard solutions for ICP-MS (Inorganic Ventures, Christiansburg, VA, United States). Recoveries were 98.3, 95.8, 97.0, and 95.0% for Fe, Mn Cu, and Zn, respectively, and limits of detection were 20, 2, 2 and 20 μg L^-1^ for Fe, Mn Cu, and Zn, respectively. Concentrations are expressed as μg metal g tissue DW^-1^.

Nicotianamine and DMA were extracted from 50 mg of ground embryo or endosperm tissue (3–4 independent replicates) with 300 μL Type I water containing 18 μL of 1 mM nicotyl-lysine -used as internal standard- following the procedure previously developed for rice seeds (Banakar et al., 2017a). The extracts were analyzed for NA and DMA using an Alliance 2795 HPLC system (Waters, Mildford, MA, United States) coupled to a time-of-flight mass spectrometer (MS-TOF; MicrOTOF, Bruker Daltonics, Bremen, Germany) equipped with an electrospray (ESI) source. For a detailed description of the method, see Banakar et al. (2017a). The limit of quantification (LOQ), defined as the concentration giving a signal to noise ratio of 10, was 1.0 μg g^-1^ tissue FW for NA and DMA. All ligand concentrations are expressed as μg NA (or DMA) g tissue FW^-1^.

### Imaging Elemental Distribution in Seed Sections

#### Sample Preparation

Thin sections (50–70 μm-thick) were obtained from fully developed, de-husked rice grains of the different genotypes using a vibrating blade microtome (VT1000 S, Leica Microsystems GmbH, Wetzlar, Germany), following the protocol described by Johnson et al. (2011). Seeds were glued (with Loctite Super Glue-3, Barcelona, Spain) to the excised bottom of a 1.5 mL plastic Eppendorf tube, and blades used were Chrome Platinum (Bic, Clichy, France). Vibratome parameters were a blade movement speed of 0.2 mm s^-1^ and a vibration frequency of 70 Hz. A 100 μm-thick Kapton polyimide film (DuPont, Des Moines, IA, United States) was used to hold the tissue section as cutting proceeded, to minimize endosperm fragmentation (Johnson et al., 2011). Longitudinal dorso-ventral seed sections were used for optical microscopy, Perl’s staining and LA-ICP-MS analysis. Sections were transferred to synchrotron adhesive tape (Leica), attached to glass slides, observed with a stereomicroscope (MZ16, Leica) and images taken with the Leica Application Suite V3.5. Sections were stored at 4°C until LA-ICP-MS analysis.

#### Perl’s Prussian Blue Staining

Seed sections (60 μm-thick) were used immediately to localize Fe using Perls staining. Sections were incubated with a solution containing 2% K_4_[Fe(CN)_6_] and 2% HCl for 15 min at room temperature. This staining technique allows for the detection of labile Fe forms, including Fe complexes with NA and citrate, Fe hydroxides and inorganic Fe, as the blue compound ferric ferrocyanide (Roschzttardtz et al., 2009; Rios et al., 2016). Stained sections were observed with a stereomicroscope (MZ16, Leica) and images taken with the Leica Application Suite V3.5.

### LA-ICP-MS Analysis

Rice seed sections (60 μm-thick) adjacent to those used for Perl’s staining were placed on synchrotron adhesive tape (Leica) and directly analyzed using a Laser Ablation (LA) system (LSX-213, Teledyne Cetac Technologies, Omaha, NE, United States) coupled to an ICP-MS instrument (Element II, Thermo Fischer Scientific, Waltham, MA, United States). Preliminary analyses were first carried out by driving LA straight lines through whole longitudinal dorso-ventral seed sections, and intense ICP-MS signals for the elements of interest were observed only in the embryo, with the endosperm providing very low or no signal. Optimized LA settings allowed for distinguishing embryo structures and obtaining signals of good intensity for the different elements, but the analysis time required for a single whole seed section was longer than 15 h. Therefore, the LA-ICP-MS analyses had to be restricted to the embryo and neighboring endosperm.

Optimized settings used for the LA-ICP-MS analysis (e.g., laser spot diameter and scan speed) are shown in **Table 1**. The net intensity of the signal obtained for each element (^31^P, ^32^S, ^55^Mn, ^56^Fe, ^63^Cu, ^64^Zn) was normalized using that of ^13^C as an internal standard for quantification purposes. Quantification of the selected elements was carried out using two certified reference materials (CRMs) for calibration: the rice flour standards NIST 1568b (National Institute of Standards and Technology, United States) and NCS ZC73028 (LGC Standards, UK). Powdered CRMs were pressed to pellets 5 mm in diameter using a laboratory press (applying 2 t for 5 min) and subsequently analyzed using the same experimental conditions optimized for the seed sections. Three ablation lines were performed for each CRM along the pellet, and the resulting normalized intensity signals, together with respective elemental concentrations were used to build calibration curves. The linear regression equations and coefficients for ^31^P, ^32^S, ^55^Mn, ^56^Fe, ^63^Cu, ^64^Zn calibration curves are shown in **Supplementary Table S1** and the calibration curve obtained for ^32^S is shown as an example in **Supplementary Figure S1**. Quantitative two-dimensional images of the elemental distributions in seed sections were created using the software packages Origin^®^ (OriginLab, Northampton, MA, United States) and ImageJ (NIH, Bethesda, WA, United States). Each data point (or pixel) was converted from intensities into concentrations, using the calibration curve for each element of interest. Then, quantitative elemental map images were first obtained by processing concentration data with the software Origin^®^. Secondly, the concentration data for Fe and Zn were also processed with the software ImageJ^®^, which permits obtaining images with a higher resolution; these maps were obtained for the WT and one line each from the three transgenic types. In the images processed with ImageJ, different concentration scales were used for each section to highlight differences in metal localization and concentration along the seed structures.

**Table 1:** Operating conditions of the laser ablation (LA) and ICP-MS devices.

LA system			ICP-MS
Laser energy	100% (∼5.6 mJ)	RF Power	1330 W
Repetition rate	20 Hz	Cooling gas	15.5 L min^-1^
Spot diameter	25 μm	Auxiliary gas	0.8 L min^-1^
Scan speed	17 μm s^-1^	Nebulizer gas (Ar)	0.8 L min^-1^
Ablation mode	single line scan	Cones	Ni (skimmer and sampler)
Carrier gas (He)	1 L min^-1^	Isotopes	^13^C, ^31^P, ^32^S, ^55^Mn, ^56^Fe, ^63^Cu, ^64^Zn
Cryogenic cell T	-20°C	Sample time	5 ms
		*Mass window*	*100%*
		*Samples per peak*	*10*


Limits of detection (LODs) were calculated by using the 3s criterion (3s_b_/S), where s_b_ is the standard deviation of 5 independent measurements of the blank value in counts per s and S is the sensitivity for the corresponding analyte isotope obtained by measuring the CRM NIST 1568b. At the selected operating conditions and using the CRM pellets, LODs were (in μg g^-1^) 0.77 for ^31^P, 11 for ^32^S, 0.10 for^55^Mn, 0.62 for ^56^Fe, 0.049 for ^63^Cu, and 0.13 for ^64^Zn, with the higher LOD for S being due to the relatively high background. The detection limits in the rice seed sections were higher, likely due to differences between the thin seed sections and the pressed pellets used for rice flour standards.

### Statistical Analysis

Statistical analysis was carried out with SPSS Statistics (v.22, IBM, New York, NY, United States) using (i) ANOVA test (*P* ≤ 0.05) to determine differences between data from transgenic and WT plants and (ii) bivariate Pearson correlation to determine whether significant linear relationships existed between the concentrations of metal ligands and metals in the embryo and endosperm.

## Results

**FIGURE 1:**
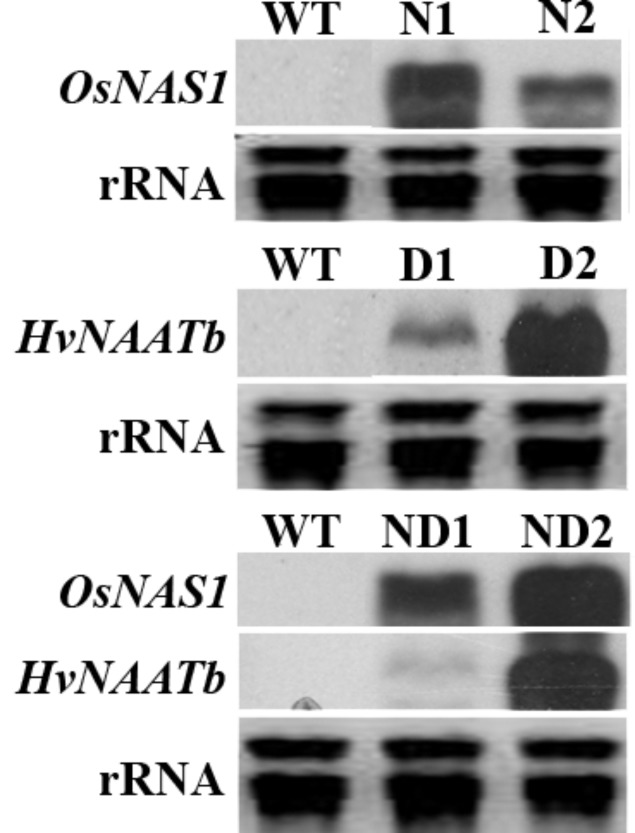
RNA blot analysis showing transgene expression in the leaf tissue of wild-type (WT) and six independent transgenic lines, two expressing *OsNAS1* (lines N1 and N2), two expressing *HvNAATb* (lines D1 and D2) and two co-expressing *OsNAS1* and *HvNAATb* (lines ND1 and ND2). rRNA, ribosomal RNA. Plants were grown under nutrient-sufficient conditions, and total RNA was isolated from flag leaves at physiological maturity.

We co-transformed rice mature seed-derived embryos with separate constructs harboring rice NAS (*OsNAS1*) and/or barley NAAT (*HvNAATb*), along with the selectable marker *hpt*. RNA blot analysis using rRNA isolated from flag leaf tissue identified lines expressing *OsNAS1* alone (N1 and N2), *HvNAATb* alone (D1 and D2) and *OsNAS1* and *HvNAATb* together (ND1 and ND2) (**Figure 1**). There was considerable variation in the transgene expression levels among the lines used: the expression of *OsNAS1* was higher in N1 than in N2, the expression of *HvNAATb* was much higher in D2 than in D1, and the expression of both *OsNAS1* and *HvNAATb* were higher in ND2 than in ND1. Brown seeds from these plants were harvested (T_4_ seeds), separated into embryo and endosperm, in both cases including their corresponding aleurone layer, and analyzed for NA, DMA and metals.

### Nicotianamine and 2′-Deoxymugineic Acid Concentrations in the Embryo and Endosperm

**FIGURE 2:**
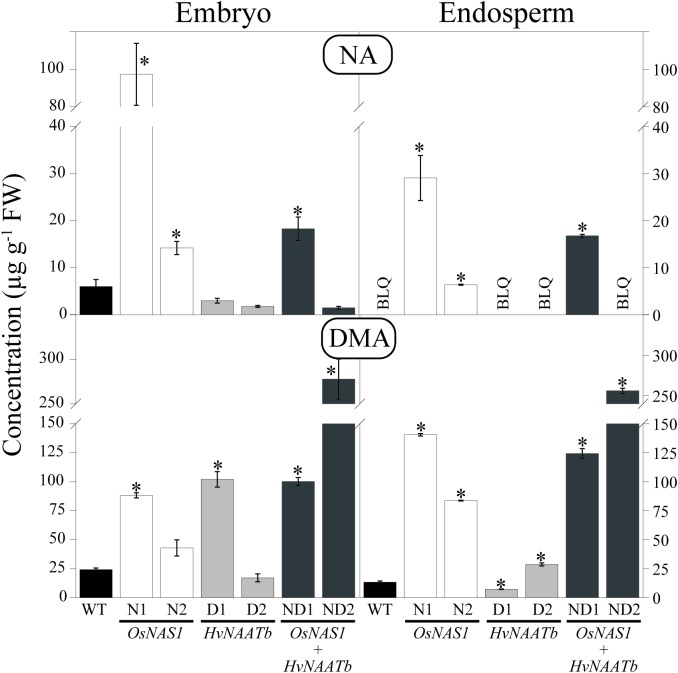
Nicotianamine (NA) and 2′-deoxymugineic acid (DMA) concentrations (in μg g^-1^ FW) in the embryo and endosperm of WT rice and six different transgenic lines, two expressing *OsNAS1* (lines N1 and N2), two expressing *HvNAATb* (lines D1 and D2) and two co-expressing *OsNAS1* and *HvNAATb* (lines ND1 and ND2). Plants (WT and T_3_ transgenic lines) were grown under nutrient-sufficient conditions, and the WT and T_4_ seeds were harvested at physiological maturity. Asterisks indicate significant differences between WT and transgenic plants as determined by Student’s *t*-test (*P* < 0.05). Values shown are means ± SE, *n* = 3–4. BLQ: below limit of quantification.

The embryo and endosperm of the WT and transgenic lines were analyzed by HPLC-ESI-MS to determine the concentrations of NA and DMA (**Figure 2**). This study is, to the best of our knowledge, the first to report NA and DMA concentrations in the rice embryo, since previous studies have only used polished and/or unpolished seeds, without analyzing embryos. In WT seeds, the NA concentration was 6.0 ± 1.5 μg g^-1^ FW in the embryo and below the LOQ (marked as BLQ in **Figure 2**) in the endosperm, whereas the DMA concentrations were 24.2 ± 1.4 and 13.5 ± 1.0 μg g^-1^ in the embryo and endosperm, respectively (**Figure 2**). Therefore, the embryo was richer in NA and DMA than the endosperm; the DMA/NA ratios were approximately 4 and very high in the embryo and endosperm, respectively (**Supplementary Figure S1**). DMA was also reported to be more abundant than NA in other rice compartments, including seeds (polished and unpolished; Masuda et al., 2009; Lee et al., 2011; Masuda et al., 2012; Trijatmiko et al., 2016), roots and leaves (Masuda et al., 2009), as well as phloem and xylem saps (Kato et al., 2010; Ando et al., 2013).

In transgenic lines, changes in NA and DMA concentrations in the embryo and the endosperm were observed when compared to the WT (**Figure 2**). The embryo NA concentrations of lines N1, N2 and ND1 were 15-, 2.5-, and 3.0-fold higher, respectively, whereas endosperm NA concentrations were well above the LOQ (29.1 ± 4.8, 6.4 ± 0.1, and 16.8 ± 0.3 μg g^-1^, respectively). In contrast, in lines D1, D2 and ND2, the embryo NA concentrations were lower than those of the WT (although not significantly at *P* ≤ 0.05). The embryo DMA concentrations of lines N1, D1, ND1 and ND2 were 3.5-, 4.2-, 4.1-, and 12-fold higher, respectively, than those in the WT. The endosperm DMA concentrations in lines N1, N2, ND1 and ND2 were also much higher than those in the WT (10.5-, 6.2-, 9.2-, and 19.0-fold, respectively), whereas they increased 2-fold in D2 and decreased by 71% in D1.

As a result of these changes, the DMA/NA ratios in the embryo of N1 and N2 (1 and 3, respectively) were lower than those in the WT (**Supplementary Figure S1**). Conversely, in D1, D2, ND1, and ND2 the DMA/NA ratios in the embryos were higher than in the WT, 34, 10, 7 and 185, respectively. On the other hand, the DMA/NA ratios in the endosperm were very high in D1, D2, and ND2 (in these three lines the NA concentrations were below the LOQ), whereas in N1, N2 and ND1 they were much lower, 5, 13 and 7, respectively.

### Metal Micronutrient Concentrations in the Embryo and Endosperm

**FIGURE 3:**
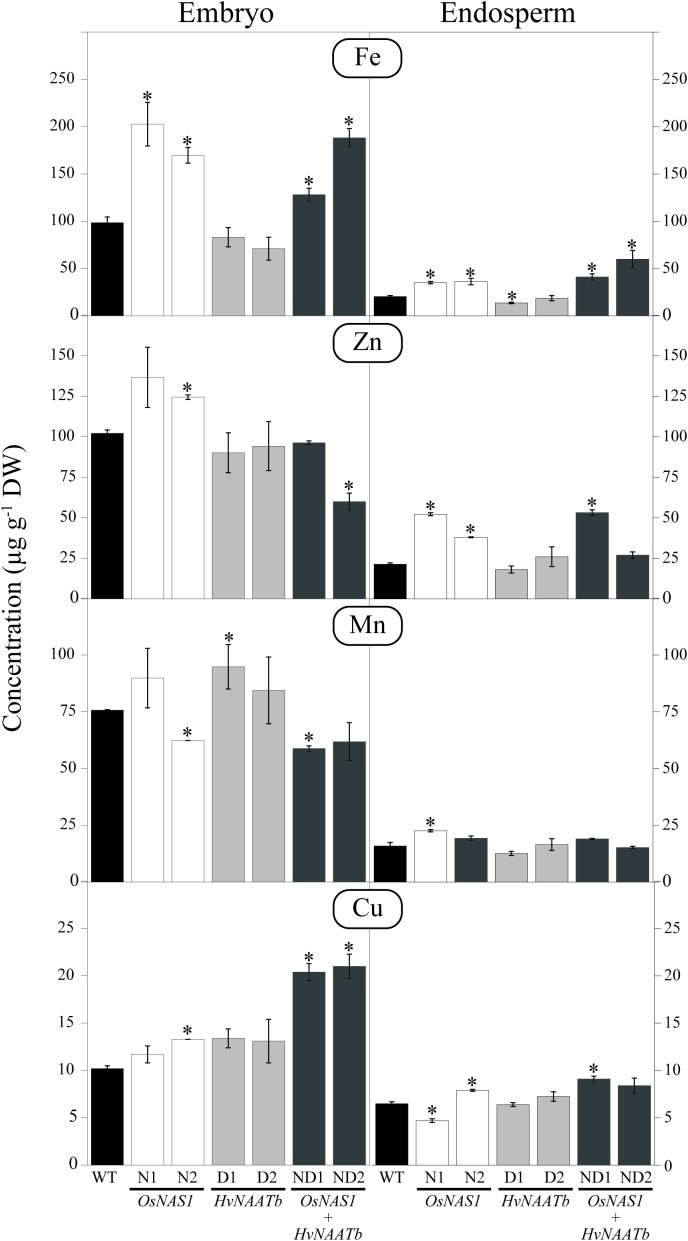
Micronutrient metal concentrations (μg g^-1^ DW) in embryo and endosperm of WT rice and six different transgenic lines, two expressing *OsNAS1* (lines N1 and N2), two expressing *HvNAATb* (lines D1 and D2) and two co-expressing *OsNAS1* and *HvNAATb* (lines ND1 and ND2). Plants (WT and T_3_ transgenic lines) were grown under nutrient-sufficient conditions. WT and T_4_ seeds were harvested at physiological maturity. Asterisks indicate significant differences between WT and transgenic plants as determined by Student’s *t*-test (*P* < 0.05). Values shown are means ± SE, *n* = 3. BLQ: below limit of quantification.

The embryo and endosperm of the WT and transgenic lines were analyzed by ICP-MS to determine the concentrations of Fe, Zn, Mn, and Cu (**Figure 3**). In the WT, the Fe concentrations were 99 ± 6 and 20 ± 1 μg g^-1^ DW in the embryo and endosperm, respectively. In lines N1, N2, ND2, and ND1 the Fe concentrations were significantly higher both in the embryo (1.3- to 2.1-fold increases over the WT values) and the endosperm (1.7- to 2.9-fold increases). In contrast, in D1 and D2 the Fe concentrations in the embryo and endosperm were not affected significantly, with the exception of a 33% decrease in the endosperm of D1.

The Zn concentrations in the WT were 102 ± 2 and 21 ± 1 μg g^-1^ in the embryo and endosperm, respectively (**Figure 3**). In the embryo, Zn concentrations increased significantly over the WT values in N1 (1.3-fold) and N2 (1.2-fold), whereas they decreased by 40% in ND2. In the endosperm, Zn concentrations increased over the WT values in N1 (2.5-fold), N2 (1.8-fold), and ND1 (2.5-fold).

The Mn concentrations in the WT were 76 ± 1 and 16 ± 2 μg g^-1^ in the embryo and endosperm, respectively (**Figure 3**). Significant changes in the Mn concentration in the embryo were only observed in N2, ND1 (a 18% decrease in both lines) and D1 (a 1.3-fold increase). In the endosperm, the only significant change in Mn concentration was found in N1, which showed a 1.4-fold increase when compared to the WT.

The Cu concentrations in the WT were 10 ± 1 and 7 ± 1 μg g^-1^ in the embryo and endosperm, respectively (**Figure 3**). In the embryo, the only significant changes in Cu concentrations were in N2 (a 1.3-fold increase), and ND1 and ND2 (2-fold increases). In the endosperm, the only changes found in Cu concentrations were in N1 (a 27% decrease), and N2 and ND1 (1.2 and 1.4-fold increases, respectively).

### Correlations Between NA, DMA and Metal Micronutrients in the Embryo and Endosperm

The relationships between the concentrations of metal ligands and metals in the embryo and endosperm were studied by bivariate Pearson correlation analysis, using all data (in nmol g^-1^ DW) from the WT and transgenic lines (**Supplementary Table S2**). Correlations found were different in the endosperm and embryo. In the embryo, DMA was positively correlated with Cu (*r* = 0.610; *P* ≤ 0.05), whereas NA was positively correlated with Fe and Zn (*r* = 0.643 and 0.647, respectively; in both cases significant at *P* ≤ 0.05). In the endosperm, there were highly significant and strong positive correlations between DMA and Fe (*r* = 0.702; *P* ≤ 0.01) and DMA and NA (*r* = 0.943; *P* ≤ 0.01), whereas no significant correlation was found between DMA and other metals (**Supplementary Table S2**). There was no correlation between NA and metals, whereas several correlations between metals were found, including Mn *vs.* Zn (*r* = 0.782; *P* ≤ 0.01), Mn vs. Cu (*r* = 0.725; *P* ≤ 0.01) and Fe vs. Zn (*r* = 0.566; *P* ≤ 0.05). Correlations between Fe and Zn have been found in previous studies (Lombi et al., 2011; Anuradha et al., 2012; Banakar et al., 2017b; Kampuang et al., 2017), and are expected due to the fact that both metals share mechanisms for uptake, short- and long-distance transport in the plant and intracellular trafficking.

In some cases, parameters measured in the embryo showed correlations with those measured in the endosperm (**Supplementary Table S2**). There were strong positive correlations between NA in the endosperm and DMA in the embryo (*r* = 0.986; *P* ≤ 0.01), and negative correlations between endosperm Fe and Cu, and embryo Cu (*r* = -0.644 and -0.604, respectively; in both cases at *P* ≤ 0.05).

### Perl’s Staining of Rice Seed Sections

**FIGURE 4:**
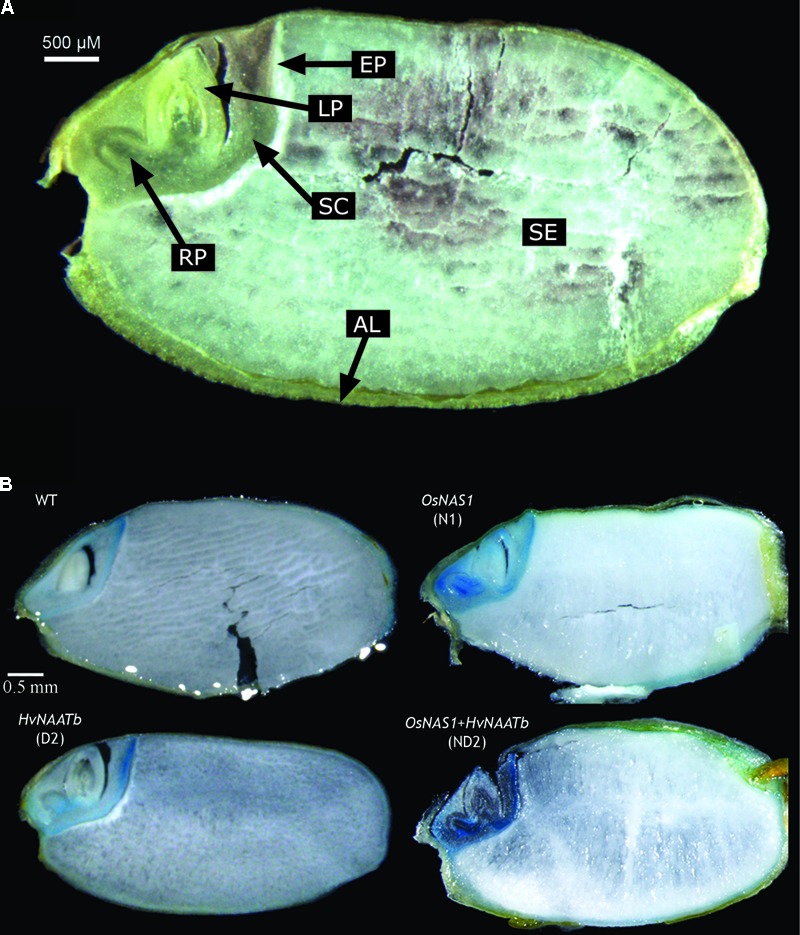
Images of longitudinal dorso-ventral sections of a rice seed. Optical image describing the different structures: AL, aleurone layer; LP, leaf primordium; RP, root primordium; SC, scutellum; SE, starchy endosperm; EP, epithelium **(A)**. Perl’s Prussian blue staining of WT rice seeds and three different transgenic lines, one expressing *OsNAS1* (line N1), one expressing *HvNAATb* (line D2) and one co-overexpressing *OsNAS1* and *HvNAATb* (line ND2) **(B)**. Plants (WT and T_3_ transgenic lines) were grown under nutrient-sufficient conditions. WT and T_4_ seeds were harvested at physiological maturity.

The Perl’s staining of longitudinal dorso-ventral sections of WT and transgenic seeds reveal the accumulation of Fe in the embryo region and the aleurone layer, whereas the blue color was absent in the endosperm (an optical image of the WT seed is shown in **Figure 4A**, and the Perl’s stain is shown in **Figure 4B**). The distribution of Fe in the embryo differed among genotypes studied. In the WT, Fe was accumulated mainly in the epithelium, and at lower levels in the scutellum and some parts of the aleurone layer. In N1 (overexpressing only *OsNAS1*) the whole embryo had more labile Fe than the WT. This increase in labile Fe was marked in the root primordia and scutellum, and also visible in the epithelium, the tip of the leaf primordia and the aleurone layer that covers the embryo and endosperm. While the pattern of Fe over-accumulation was similar among lines overexpressing *OsNAS1*, in ND2 (co-expressing *OsNAS1* and *HvNAATb*) there was a much higher Perl’s stain than in N1, when *OsNAS1* was overexpressed alone. In D2 (expressing only *HvNAATb*) there was an over-accumulation of labile Fe in specific areas of the embryo, mainly in the root primordia and scutellum, although this effect was much less intense than in N1, whereas the Perl’s stain in the epithelium and aleurone layer did not differ from the WT.

### Quantitative Images of the Elemental Distributions in Rice Seed Sections Obtained by LA-ICP-MS

**FIGURE 5:**
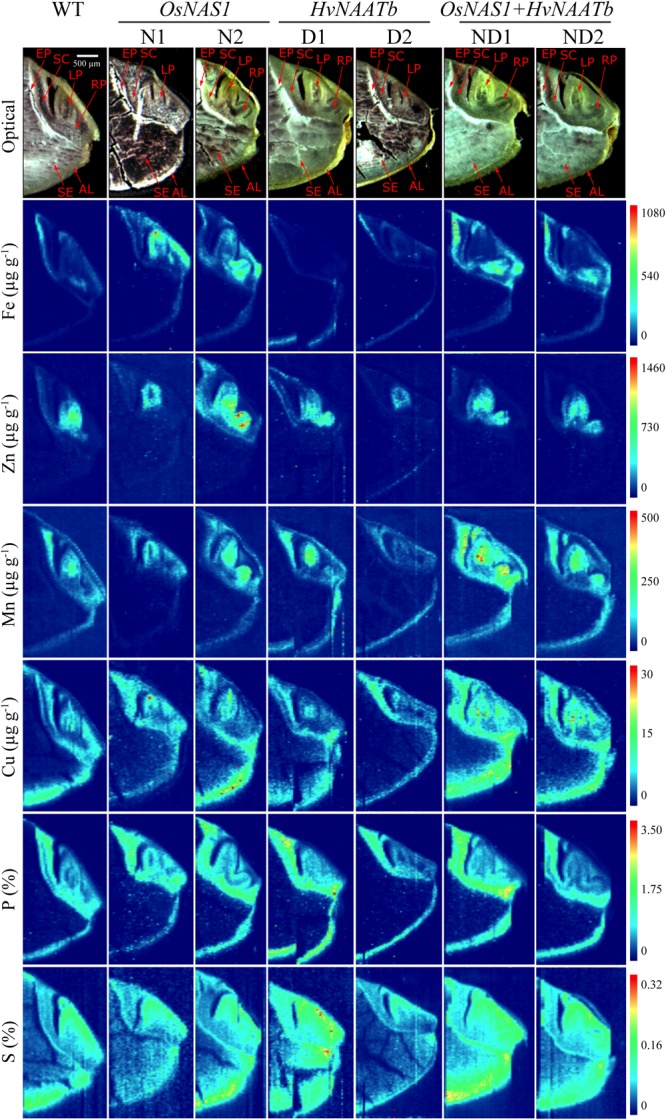
Quantitative images obtained by LA-ICP-MS for Fe, Zn, Mn, Cu, P, and S distribution in the embryo and neighboring endosperm tissues for WT rice and six different transgenic lines, two overexpressing *OsNAS1* (lines N1 and N2), two expressing *HvNAATb* (lines D1 and D2) and two co-expressing *OsNAS1* and *HvNAATb* (lines ND1 and ND2). Plants (WT and T_3_ transgenic lines) were grown under nutrient-sufficient conditions, and the WT and T_4_ seeds were harvested at physiological maturity and dehusked. 60 μm-thick seed sections were used for LA-ICP-MS analysis. Elemental images were obtained processing LA-ICP-MS data with the software Origin^®^. Color scales represent the concentrations for each element, with the lowest ones in dark blue and the highest ones in red. Depending on the element, the scale bar indicates the elemental concentrations in μg g^-1^ (Fe, Zn, Mn, and Cu) and in mass fraction percentage (S and P). For a given element, the same scale was used in all genotypes. Pictures shown in the first row are optical images of the sections subjected to LA-ICP-MS analysis. AL, aleurone layer; LP, leaf primordium; RP, root primordium; SC, scutellum; SE, starchy endosperm; EP, epithelium.

**FIGURE 6:**
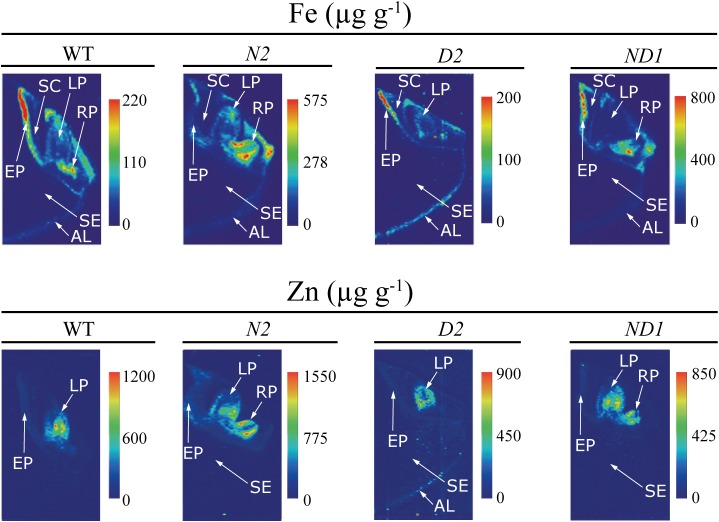
High-resolution quantitative images obtained by LA-ICP-MS of Fe and Zn in the embryo and neighboring endosperm tissues of WT rice and three different transgenic lines, one expressing *OsNAS1* (line N2), one expressing *HvNAATb* (line D2) and one co-expressing *OsNAS1* and *HvNAATb* (line ND1). Quantitative images of Fe and Zn distributions were obtained processing LA-ICP-MS data with the software ImageJ^®^. The scale bar indicates the elemental concentrations in μg g^-1^, with the maximum in red and the minimum in dark blue. The concentration scale for each image is different. AL, aleurone layer; LP, leaf primordium; RP, root primordium; SC, scutellum; SE, starchy endosperm; EP, epithelium.

The quantitative two-dimensional images obtained for Fe, Zn, Mn, Cu, P and S distribution in the seeds are shown in **Figures 5**, **6**, with the lowest and highest concentrations being represented in dark blue and red, respectively. First, maps are presented together with the corresponding optical images of the same sections, using the same scale for all genotypes (**Figure 5**). The match between elemental maps and optical images allows for the allocation of elemental concentrations to the different embryo structures. Also, high resolution maps were drawn using different concentration scales for each sample to depict optimal contrast (**Figure 6**).

This is the first time quantitative LA-ICP-MS elemental map images (Fe, Zn, Mn, Cu, P, and S) have been obtained for the embryo structures of WT and biofortified rice seeds. Previous studies applying LA-ICP-MS imaging to rice seeds did not provide quantitative images for Fe, and those for Zn had only a low resolution (Wirth et al., 2009; Basnet et al., 2014, 2016). The first remarkable aspect in the elemental distribution was the preferential accumulation of most elements in the embryo, with the element concentrations in the endosperm being generally lower, with the only exceptions of Cu and S (**Figure 5**). This is in agreement with previous results reported by using semi-quantitative, high-resolution techniques such as synchrotron-based X-ray fluorescence spectroscopy (Lombi et al., 2009; Takahashi et al., 2009; Wirth et al., 2009; Johnson et al., 2011; Iwai et al., 2012; Lu et al., 2013; Kyriacou et al., 2014) and secondary ion mass spectrometry (Kyriacou et al., 2014). Since most seed parts are quite heterogeneous, their composition are best described in terms of concentration ranges for each element.

In the case of Fe, there were large differences in concentrations between seed parts, with the distribution being quite different in the WT and some of the transgenic lines, with the exception of the endosperm, where Fe concentrations were below 10 μg Fe g^-1^ for all genotypes (**Figures 5**, **6**). In the WT, the highest Fe concentrations were found in the epithelium and root primordium (70–300 μg Fe g^-1^), whereas lower concentrations were found in the aleurone layer (10–140 μg Fe g^-1^), leaf primordium (25–100 μg Fe g^-1^) and scutellum (25–75 μg Fe g^-1^) (**Figures 5**, **6**). In N1 and N2, the Fe concentration was increased in the scutellum (to the range 150–650 μg g^-1^ in both lines), leaf primordium (150–950 and 150–350 μg g^-1^ in N1 and N2, respectively), root primordium (150–400 and 150–600 μg g^-1^ in N1 and N2, respectively) and aleurone layer (70–300 μg Fe g^-1^ in both lines) (**Figure 5**; see also N2 in **Figure 6**). Lines D1 and D2 showed lower Fe concentrations in most embryo tissues than those found in the WT, with D1 being more affected than D2 (**Figure 5**; see also D2 in **Figure 6**). Iron concentrations decreased in D1 and D2 in the scutellum (below 50 μg g^-1^) and the root primordium (<50 μg g^-1^ and to 50–100 μg g^-1^ in D1 and D2, respectively), whereas decreased in the leaf primordium and epithelium only in D1 (<50 and 50–100 μg g^-1^, respectively) and increased in the aleurone layer only in D1 (50–250 μg g^-1^). Lines ND1 and ND2 showed large Fe concentration increases in the epithelium (200–900 μg g^-1^; **Figure 5**; see also ND1 in **Figure 6**), compared to the values found in the WT and the other four transgenic lines. Other relevant changes in Fe distribution in ND1 and ND2 were: (i) increases in root primordium Fe concentrations over those in the WT but not always over those in N2 (up to 200–900 and 100–400 μg g^-1^, respectively), (ii) increases in scutellum Fe concentrations over those in the WT but only slightly higher than those in N1 and N2 (150–550 and 100–350 μg g^-1^, respectively), and (iii) decreases in the leaf primordium Fe concentrations (<50 μg g^-1^) below those in the WT.

In the case of Zn, large differences in concentrations were also observed between the different seed parts, with modifications in the distribution in the transgenic genotypes when compared to the WT, whereas Zn could not be detected in the endosperm of any genotype (**Figures 5**, **6**). The WT seed showed the highest Zn concentration in the leaf primordium (500–1100 μg g^-1^), followed by the scutellum and epithelium (50–200 μg g^-1^) and the aleurone layer (<100 μg g^-1^). Lines N1 and N2 showed lower concentrations of Zn than those found in the WT in the leaf primordium (300–800 and 50–1100 μg g^-1^, respectively), whereas in the root primordium, scutellum, epithelium and aleurone layer the Zn concentrations increased only in N2 (50–1500, 200–550, <500 and <200 μg g^-1^, respectively) when compared with the WT. In D1 and D2, Zn concentrations in both the leaf primordia (<600 and 200–500 μg g^-1^, respectively) and scutellum (50–100 and <75 μg g^-1^, respectively) were lower than those in the WT. Other changes in Zn distribution when compared with the WT were different for these two lines: Zn increased in D1 in the root primordia and epithelium (500–1000 and 150–350 μg g^-1^, respectively) and decreased in D2 (<75 μg g^-1^ in both tissues). In ND1 and ND2 Zn concentrations in the leaf primordia (300–700 μg Zn g^-1^, respectively) were lower than those in the WT, and similar to those found in N1 and N2. Also, moderate increases in the Zn concentrations in the root primordium and scutellum were observed in the two double transgenic lines, especially when compared with those in N1.

Manganese could not be detected in the endosperm of any genotype, and was generally localized in high concentrations in the leaf and root primordia in all genotypes (100–400 and 100–130 μg g^-1^, respectively), with concentrations in the epithelium and aleurone layer being also similar (<40 and 50–150 μg g^-1^, respectively; **Figure 5**). The only exception was ND1, which showed higher Mn concentrations in all tissues (100–500 μg Mn g^-1^; **Figure 5**).

Copper was the less abundant micronutrient of those investigated, and differences in distribution between lines were less marked (**Figure 5**). In the WT, this element was mostly found in the aleurone layer, scutellum and leaf primordium (concentrations in the range of 5–15 μg Cu g^-1^), with Cu concentrations in the rest of the tissues being ≤10 μg g^-1^, with the exception of the inner endosperm, where Cu could not be detected. In N1, N2, ND1 and ND2 some changes in Cu distribution were observed: increases in Cu concentrations in the root primordia (9–12, 8–25, 10–30 μg g^-1^ in N1, ND1 and ND2), leaf primordia (9–40 μg g^-1^ in N1), and aleurone layer (10–30, 8–25, 8–25 μg g^-1^ in N2, ND1 and ND2). In contrast, in D1 and D2 no changes were observed in the Cu distribution.

Phosphorus was present with similar distribution and concentrations in all genotypes used (**Figure 5**). This element was mainly located in the embryo and the aleurone layer (at concentrations between 1 and 2%) and was not detected in the endosperm. Sulfur was present in the whole embryo and the endosperm, and it was mainly located in the leaf and root primordia and the aleurone layer (at concentrations of approximately 0.2%), without any consistent difference among genotypes (**Figure 5**). It is also worth to remark the gradient of S concentrations, from high in the aleurone layer to low in the endosperm.

## Discussion

**FIGURE 7:**
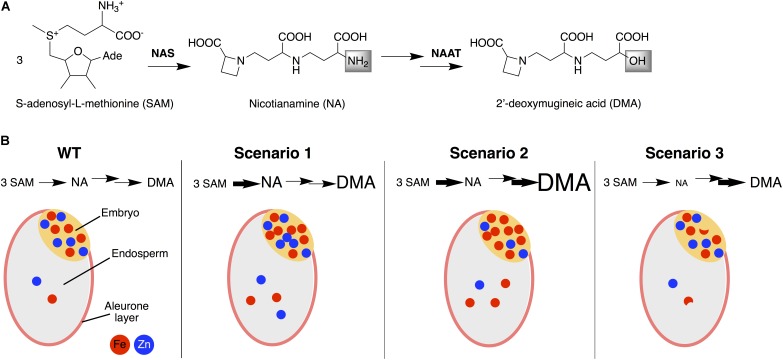
Simplified biosynthesis pathway of nicotianamine (NA) and 2′-deoxymugineic acid (DMA) **(A)** and a schematic representation of the accumulation and distribution of Fe and Zn between the embryo and endosperm of rice seeds in WT and in the three DMA/NA scenarios studied **(B)**. Scenario 1 occurs (e.g., in N1, N2 and ND1) when an enhanced NA level is not fully depleted because of a limited capacity to use NA for DMA synthesis, resulting in consistent enrichments in NA, DMA, Fe, and Zn in the endosperm and NA, DMA and Fe in the embryo. Scenario 2 occurs (e.g., in ND2) when an enhanced NA level is depleted by an enhanced capacity use NA for DMA synthesis, resulting in enrichments only for DMA and Fe, both in the endosperm and embryo. Scenario 3 occurs (e.g., in D1 and D2) when the lack of sufficient NA replenishment limits DMA synthesis, in spite of the enhanced capacity to use NA for this purpose, and results in decreases in NA, variable changes in DMA and moderate decreases in Fe in the embryo and endosperm. NAS, nicotianamine synthase; NAAT, nicotianamine aminotransferase.

Enhancing NA synthesis *via* genetic transformation has been shown to increase the concentrations of Fe and Zn in rice seeds (Nozoye, 2018). However, it was still not known whether this was due to the enrichment in NA itself or to the subsequent enrichment in its derivative DMA, and whether the changes in the relative levels of both ligands may have an effect on the partitioning of metals between the embryo and endosperm. In this study we analyzed the concentrations of NA, DMA and metals in the embryo and endosperm of WT rice and six transgenic lines, overexpressing *OsNAS1* and/or expressing barley *NAAT* (*HvNAATb*), which provided contrasting levels of DMA and NA. This allows for outlining three different DMA/NA scenarios for metal distribution in the rice seed (**Figure 7**) that are discussed below. Results show that increasing DMA alone led to Fe enrichment in the embryo and endosperm, whereas increasing DMA in combination with NA led to Fe and Zn enrichment in both tissues (**Figure 7**).

### First DMA/NA Scenario

In a first scenario, an enhanced NA level would not be fully depleted because of the limited capacity to use NA for DMA synthesis (**Figure 7**). Lines complying with this scenario were those having an enhanced expression of *OsNAS1* alone (N1 and N2), or in combination with a low expression of barley *NAATb* (ND1), and showed consistent enrichments in NA, DMA, Fe and Zn in the endosperm, and also to enrichments of NA, DMA and Fe in the embryo (**Figures 1–3**). In the endosperm of these lines, the increases in DMA and NA concentrations (6- to 10-fold and 6- to 29-fold, respectively) were much larger than those found for Fe and Zn (1.7- to 2.0-fold and 1.7- to 2.5-fold, respectively), and occasionally accompanied of moderate changes in other metals (e.g., a 1.4-fold increase for Cu in line ND1). Other rice transgenics complying with this DMA/NA scenario are those overexpressing *OsNAS1-3* (Lee et al., 2009b) or *HvNAS1* alone (Masuda et al., 2009), as well as *OsNAS2* in combination with *SoyferH1* (Trijatmiko et al., 2016), which showed concentration increases in polished seeds (endosperm) of 2- to 33-fold for DMA, 5- to 32-fold for NA, 2.0- to 7.5-fold for Fe and 2.2- to 3.8-fold for Zn. The same scenario occurs when overexpressing *OsNAS1* in combination with *HvNAATb* (Banakar et al., 2017b), leading to increases in polished seeds of 33-, 160-, 4.0-, and 4.1-fold for DMA, NA, Fe, and Zn concentrations, respectively; that study also shows an increased abundance of NA and DMA in leaves and roots, which promotes Fe and Zn uptake, root-to-shoot translocation, and finally seed loading. Other transgenics also showed concomitant Fe and Zn concentration increases, but the DMA and/or NA concentrations were not determined (Wirth et al., 2009; Boonyaves et al., 2017; Wu et al., 2018).

The elemental images of these transgenic lines (N1, N2, and ND1) confirmed that embryos were enriched in Fe, and also showed changes in the metal distribution pattern, with increases in Fe concentrations in the leaf primordium, scutellum and root primordium (**Figures 5**, **6**). The mobilization of Fe and Zn in the rice seed involves transport from the endosperm near the embryo to the epithelium, scutellum and then to the leaf and root primordia (Takahashi et al., 2009). In N1 and N2 embryos, the increases in NA and DMA concentrations (**Figure 2**) and low DMA/NA ratios (**Supplementary Figure S2**) would allow for the formation of Fe(II)-NA in addition to Fe(III)-DMA, with Fe transport occurring *via* YSLs such as OsYSL9/OsYSL2. OsYSL9 works with Fe(II)-NA and Fe(III)-DMA and is expressed in the endosperm adjacent to the embryo and scutellum (Senoura et al., 2017), whereas OsYSL2 functions with Fe(II)-NA [not with Fe(III)-DMA] and is expressed during seed development in the whole embryo (Koike et al., 2004) and in mature seeds in the epithelium, vascular bundle of the scutellum and leaf primordium (Nozoye et al., 2007).

In contrast, the embryos of ND1 were also enriched in DMA and NA (**Figure 2**) but had a slight increase in the DMA/NA ratio (**Supplementary Figure S2**). These embryos also showed an accumulation of Fe in the epithelium, scutellum and root primordium, but Fe in the leaf primordium was reduced (**Figures 5**, **6**). Since in this genotype Fe(III)-DMA would be favored over Fe(II)-NA, this reduction suggests that Fe transport to the leaf primordium occurs as Fe(II)-NA *via* OsYSL2, a transporter specific for Fe (and Mn) complexes with NA (but not with DMA) localized in the embryo (Koike et al., 2004).

The Zn distribution pattern in the embryo was also altered in this scenario when compared to the WT (**Figures 5**, **6**), especially in the leaf primordium, the structure with the highest Zn concentration. In the N1 and ND1 embryos, the extremely high levels of DMA + NA (6- and 4-fold higher than in the WT, respectively) resulted in Zn depletion not only of the leaf primordium, but also the root primordium when NA was as abundant as DMA (in N1) (**Figures 5**, **6**). In contrast, the slight increase of DMA+NA in N2 (1.4-fold) resulted in smaller decreases in the Zn concentrations in the leaf primordium and increased Zn concentrations in the scutellum and root primordium. A large abundance of Zn chelators would diminish the pool of free Zn(II) ions, therefore limiting its availability for transport *via* OsZIP4 and/or OsIRT1 throughout the embryo, and more specifically toward the meristematic tissues where this metal tends to accumulate massively. OsZIP4 is expressed in the vascular bundle of the scutellum and the leaf and root primordium (Takahashi et al., 2009, 2011), whereas OsIRT1, which transports Zn in addition to Fe (Lee and An, 2009), is also expressed in embryo structures (Nozoye et al., 2007).

### Second DMA/NA Scenario

In a second DMA/NA scenario, an enhanced NA level would be depleted by an enhanced capacity to use NA for DMA synthesis (**Figure 7**). The line complying with this scenario, ND2, had an enhanced expression of *OsNAS1* in combination with a high expression of *HvNAATb*, and showed enrichments only for DMA and Fe, both in the endosperm and embryo, whereas NA and Zn concentrations in the embryo decreased (by 76 and 40%, respectively) and Cu concentrations increased (2.0-fold), without any change in the endosperm concentrations of NA, Zn, Mn and Cu (**Figures 1–3**). In this line, the endosperm enrichments for DMA and Fe (19- and 2.9-fold, respectively) were much larger than those found in the lines of the first scenario (see above). This scenario also occurs with the constitutive expression of the strict Fe(III)-DMA transporter HvYS1 (Murata et al., 2006), which leads to increases in the concentrations of both DMA (2.3-fold) and Fe (2.1-fold) in polished seeds, without affecting the concentrations of NA, Zn and Mn (Banakar et al., 2017a). In a fully opposite DMA/NA scenario, the *Osnaat1* mutant shows a large increase in NA accompanied by a large DMA depletion, resulting in an stimulated Fe(II) acquisition and a seed enrichment in Fe (1.8- and 3.8-fold increases in unpolished and polished seeds, respectively) but not Zn (Cheng et al., 2007).

The elemental images of ND2 seeds confirms that embryos were markedly enriched in Fe, and also showed changes in the metal distribution pattern, with an accumulation of Fe in the epithelium, scutellum and root primordium, and a depletion of Fe in most of the leaf primordium (**Figure 5**). This supports that the transport of Fe from the endosperm near the embryo to the epithelium, scutellum and root primordium can be *via* OsYSL9, mediated by Fe(III)-DMA in addition to Fe(II)-NA, since the extremely high levels of DMA and major depletion of NA (**Figure 2**) would strongly favor the formation of Fe(III)-DMA over Fe(II)-NA. On the other hand, the ND2 data also provide further support to the idea that the transport of Fe from the scutellum to the leaf primordium occur as Fe(II)-NA *via* OsYSL2.

The decrease in Zn and increase in Cu in the embryo in ND2 provides some hints on the partitioning of both metals in the rice seed. The possible Zn transport forms within the rice grain include free Zn(II) ions, Zn(II)-NA, and Zn(II)-DMA, with the latter being unlikely to be relevant, since the *Osnaat1* mutant shows no Zn partitioning phenotype in the grain (Cheng et al., 2007). In a previous study, the activation of *OsNAS2* generated a new pool of bio-available Zn in the endosperm, mainly composed of Zn(II)-NA and Zn(II)-DMA (Lee et al., 2011). The excess of DMA in the embryo of ND2 may favor the formation of Zn(II)-DMA, hampering the transport of free Zn(II) ions from the endosperm *via* the highly selective transporter OsZIP4 (Ishimaru et al., 2005), expressed in the endosperm region adjacent to the epithelium in mature rice seeds (Takahashi et al., 2009). On the other hand, the excess of DMA and the depletion of NA in ND2 will difficult the formation of Zn(II)-NA, hampering transfer *via* YSLs using this metal chelate. OsYSL15 and OsYSL9 have not been assayed yet in this respect (Inoue et al., 2009; Lee et al., 2009a; Senoura et al., 2017), whereas others, including OsYSL2 (Koike et al., 2004), OsYSL16 (Zheng et al., 2012) and OsYSL18 (Aoyama et al., 2009) do not transport Zn(II)-NA. The Zn distribution pattern in the embryo was also altered in this scenario when compared to the WT (**Figure 5**), mainly affecting the leaf primordium, the structure with the highest Zn concentration. In the ND2 embryos, the extremely high levels of Zn chelators (DMA + NA) (10-fold higher than in the WT) resulted in a Zn depletion in the leaf primordium (**Figure 5**). As indicated above for N1 and ND1 (first DMA/NA scenario), the abundance of Zn chelators would tend to decrease the pool of free Zn(II) ions, therefore limiting transport *via* OsZIP4 and/or OsIRT1.

Copper in the embryo was increased in ND2, conversely to what occurs with Zn. In this second scenario, which includes high DMA and low NA availability, Cu complexation is favored, since the stability constants are higher for Cu [18.7 for Cu(II)-DMA, Murakami et al., 1989; 18.6 for Cu(II)-NA, Beneš et al., 1983] than for Zn [12.7 for Zn(II)-DMA, Murakami et al., 1989; 15.4 for Zn(II)-NA, Anderegg and Ripperger, 1989]. A likely candidate for Cu delivery to the embryo is OsYSL16, which is highly expressed in all tissues of developing seeds (Lee et al., 2012), and transports Cu(II)-NA and Fe(III)-DMA, but not Cu(II)-DMA, Fe(II)-NA and Zn(II)-NA (Kakei et al., 2012; Zheng et al., 2012). It is also possible that YSL2, YSL9 and YSL18, which are expressed in embryo and/or endosperm during seed development (Koike et al., 2004; Aoyama et al., 2009; Senoura et al., 2017), could be responsible for Cu delivery to the embryo, since YSLs can transport a broad range of substrates [for instance, ZmYS1 transports Fe(III)-DMA, Zn(II)-DMA, Cu(II)-DMA, Fe(II)-NA, Ni(II)-NA and others; Schaaf et al., 2004; Murata et al., 2006].

### Third DMA/NA Scenario

In the third DMA/NA scenario, the lack of sufficient NA replenishment would limit DMA synthesis, in spite of the enhanced capacity to use NA for this purpose (**Figure 7**). Lines complying with this scenario were those expressing *HvNAATb* alone (D1 and D2), and resulted in decreases in NA, variable changes in DMA and moderate decreases in Fe in the embryo and endosperm (**Figures 1–3**). A low expression of *HvNAATb* (D1) led to moderate decreases in DMA (26%) and Fe (35%) in the endosperm and to an accumulation of Fe in the aleurone layer (**Figure 5**), whereas in the embryo NA and Fe also decreased moderately (17 and 24%, respectively, in both cases significantly at *P* ≤ 0.10), DMA increased (3.9-fold) and Fe was depleted in all embryo structures (**Figure 5**). The presence of low levels of DMA in the endosperm would make more difficult to compete with phytic acid present in the aleurone layer, since Fe(III)-DMA and Fe(III)-phytic acid have similar stability constants (18.4 and 18.2, respectively; Murakami et al., 1989; Torres et al., 2005), and consequently, Fe would stay in the aleurone layer, limiting its transport to the inner endosperm and subsequently to the embryo. The high *HvNAATb* expression in D2 caused moderate increases in DMA (1.9-fold) in the endosperm and moderate decreases in NA and Fe in the embryo, with the Fe distribution pattern being unaffected.

## Conclusion

When the transgenic approach results in increases in the DMA concentration alone or in combination with NA (second and first DMA/NA scenarios, respectively), the prevalent mechanisms appear to be those based on Fe(III)-DMA, which enhance Fe transport and storage in the endosperm, likely using YSL transporters. When increases in DMA occur in combination with NA increases (first DMA/NA scenario), an additional mechanism based on Zn(II)-NA appears to be elicited, which boosts Zn transport and storage in the endosperm. However, when the transgenic approach results only in minor changes in the DMA levels (third DMA/NA scenario) there are no effects on the metal status in the seed. This knowledge can help designing future strategies for biofortification strategies in rice, using the selectivity of the different ligands and transporters. It should be kept in mind that in high-NA/DMA grains the bioavailability of Fe for mammals and humans is improved even when the Fe concentrations are unchanged (Zheng et al., 2010; Eagling et al., 2014). Our study demonstrates that a better understanding of transgenic plant phenotypes, using in-depth localized quantification of the targeted nutrients and related metabolites in plant tissues, will facilitate the application of more refined strategies for biofortification of staple crops.

## Author Contributions

PC, BF, and AÁ-F conceived and designed the experiments. RB obtained the plant material. PD-B performed the HPLC-ESI-MS(TOF) analysis, obtained the seed sections, and performed Perl’s staining. SR-M and BF performed the LA-ICP-MS analysis. PD-B prepared and analyzed the results, and drafted the manuscript. TC, RP, and RB analyzed critically the results. AÁ-F, JA, BF, and PC wrote, reviewed, and edited the paper. All the authors read and approved the final manuscript.

## Conflict of Interest Statement

The authors declare that the research was conducted in the absence of any commercial or financial relationships that could be construed as a potential conflict of interest.
