# Supplementary material for: Iron and Zinc in the Embryo and Endosperm of Rice (Oryza sativa L.) Seeds in Contrasting 2′-Deoxymugineic Acid/Nicotianamine Scenarios
Source: Front Plant Sci. 2018 Aug 21;9:1190. doi: 10.3389/fpls.2018.01190 (PMC6113566; doi:10.3389/fpls.2018.01190)
Supplement: Supplementary file 1 [file Table_1.DOC]

Supplementary Material

**Iron and zinc within the embryo and endosperm of rice (*Oryza sativa* L.) seeds in contrasting by 2'-deoxymugineic acid/nicotianamine scenarios**

***Pablo Díaz-Benito^1,†^, Raviraj Banakar^2,‡^, Sara Rodríguez-Menéndez^3^, Teresa Capell^2^, Rosario Pereiro^3^, Paul Christou^2,4^, Javier Abadía^1^, Beatriz Fernández^3^ and Ana Álvarez-Fernández^1*^***

*^1^ Plant Nutrition Department, Estación Experimental de Aula Dei, Consejo Superior de Investigaciones Científicas (CSIC), Zaragoza, Spain, ^2^ Departament de Producció Vegetal i Ciència Forestal, Universitat de Lleida-Agrotecnio Center Lleida, Spain, ^3^ Department of Physical and Analytical Chemistry, Faculty of Chemistry, University of Oviedo, Oviedo, Spain, ^4^ ICREA, Catalan Institute for Research and Advanced Studies, Barcelona, Spain*

***Correspondence:**

*Ana Álvarez-Fernández*

*ana.alvarez@eaad.csic.es*

**^†,‡^Present affiliation:**

**^†^***Pablo Díaz-Benito, Universidade Católica Portuguesa,*

*CBQF – Centro de Biotecnologia e Química Fina – Laboratório Associado, Escola Superior de Biotecnologia, Porto, Portugal, ^‡^Raviraj Banakar, Department of Agronomy, Iowa State University, Ames, Iowa, USA*

The following Supplementary Data are available for this article:

**Table S1.** Linear regression equations and regression coefficients calculated for ^31^P, ^32^S, ^55^Mn, ^56^Fe, ^63^Cu, ^64^Zn calibration curves obtained by LA-ICP-MS.

**Table S2.** Pearson’s correlation coefficients between ligand and metal concentrations in the embryo and endosperm.

**Figure S1** Calibration curve for sulphur obtained by LA-ICP-MS using rice certified reference materials.

**Figure S2** DMA/NA ratios in the embryo and endosperm.

**Table S1.** Linear regression equations and regression coefficients obtained for ^31^P, ^32^S, ^55^Mn, ^56^Fe, ^63^Cu, ^64^Zn calibration curves obtained by LA-ICP-MS.

| **Isotope** | **Equation –Linear Regression** | **Regression Coefficient** |
| --- | --- | --- |
| ^31^P | Int. Ratio = 18.800 [P]-0.011 | 0.978 |
| ^32^S | Int. Ratio = 44.622 [S]-0.006 | 0.999 |
| ^55^Mn | Int. Ratio = 0.011 [Mn]+0.006 | 0.985 |
| ^56^Fe | Int. Ratio = 0.015 [Fe]-0.005 | 0.993 |
| ^63^Cu | Int. Ratio = 0.009 [Cu]+0.001 | 0.990 |
| ^64^Zn | Int. Ratio = 0.003 [Zn]+0.001 | 0.995 |

**Table S2** Pearson’s correlation coefficients between the concentrations (in nmol g^-1^) of NA, DMA Fe, Mn, Cu and Zn in the embryo and endosperm.

**Figure S1.** Calibration curve obtained for ^32^S by LA-ICP-MS using two rice certified reference materials (NIST 1568b and NCS ZC73028). Y axis represents ^32^S intensity signal normalized with ^13^C.


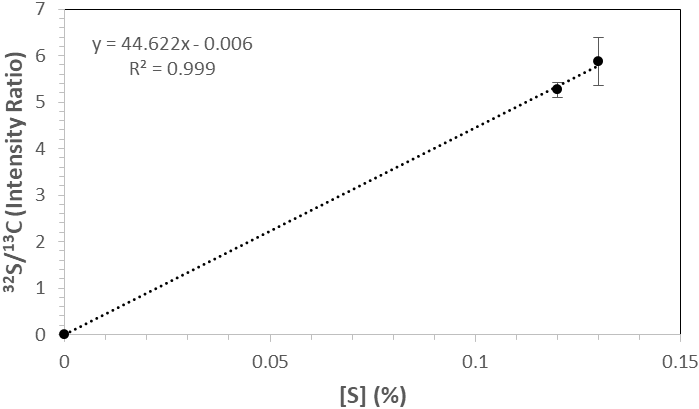


**Figure S2** 2'-deoxymugineic acid (DMA) and nicotianamine (NA) ratio in embryo and endosperm of wild-type (WT) rice and six different transgenic lines, two expressing *OsNAS1* (lines N1 and N2), two expressing *HvNAATb* (lines D1 and D2) and two co-expressing *OsNAS1* and *HvNAATb* (lines ND1 and ND2). Plants (WT and T_3_ transgenic lines) were grown under nutrient-sufficient conditions, and the WT and T_4_ seeds were harvested at physiological maturity.


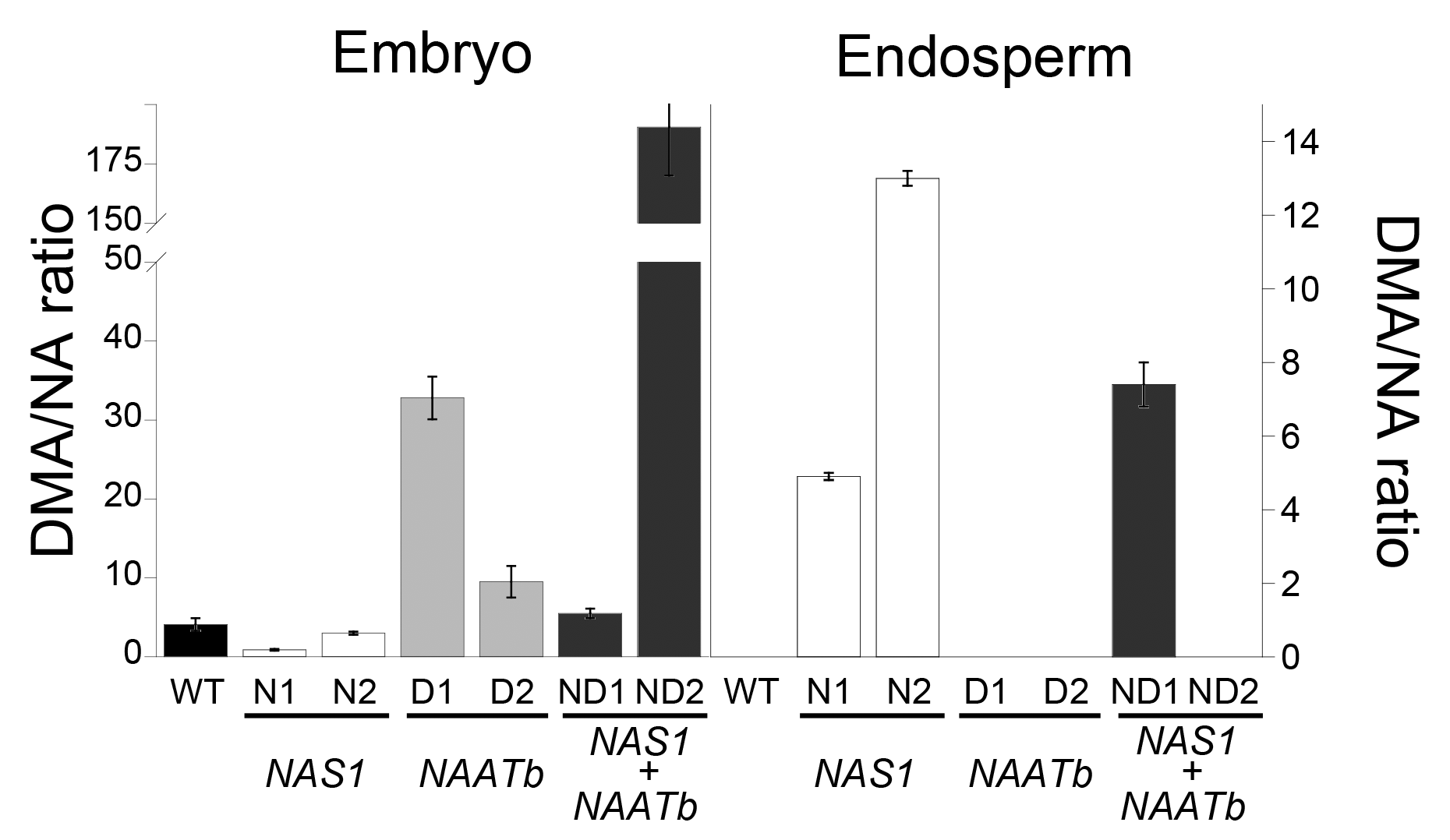


**
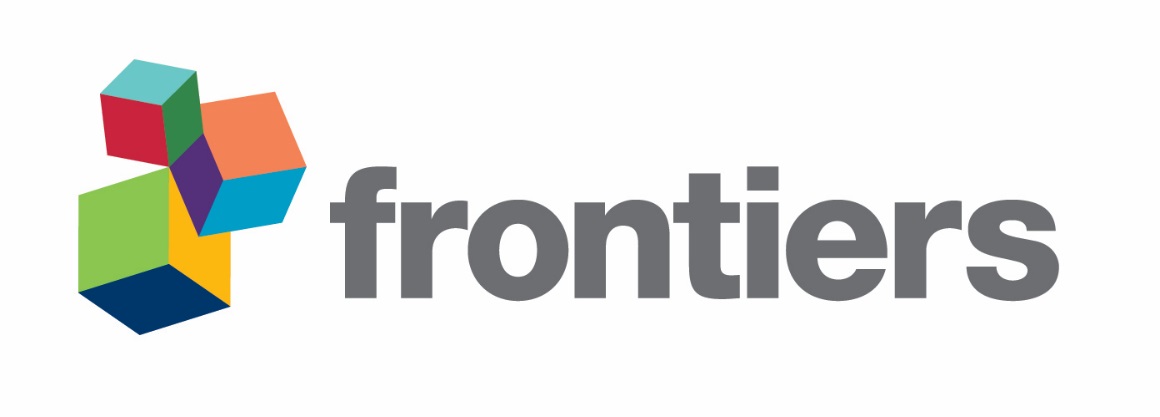
**
